# Polarization-Independent Ultra-Wideband Metamaterial Absorber for Solar Harvesting at Infrared Regime

**DOI:** 10.3390/ma13112560

**Published:** 2020-06-04

**Authors:** Asraful Alam, Sikder Sunbeam Islam, Md. Hobaibul Islam, Ali F. Almutairi, Mohammad Tariqul Islam

**Affiliations:** 1 Department of Electrical and Electronic Engineering, International Islamic University Chittagong, Chittagong 4318, Bangladesh; sikder_islam@yahoo.co.uk (S.S.I.); hobaib.eee@gmail.com (M.H.I.); 2 Department of Electrical, Electronic & Systems Engineering, Faculty of Engineering and Built Environment, Universiti Kebangsaan Malaysia, Bangi 43600, Selangor, Malaysia; 3 Space Science Centre (ANGKASA), Institute of Climate Change (IPI), Universiti Kebangsaan Malaysia, Bangi 43600, Selangor, Malaysia; 4 Electrical Engineering Department, Kuwait University, Kuwait City 13060, Kuwait

**Keywords:** metamaterial absorber, ultra-wideband, polarization independent, solar harvesting

## Abstract

This paper presents an ultra-wideband metamaterial absorber for solar harvesting in the infrared regime (220–360 THz) of the solar spectrum. The proposed absorber consists of square-shaped copper patches of different sizes imposed on a GaAs (Gallium arsenide) substrate. The design and simulation of the unit cell are performed with finite integration technique (FIT)-based simulation software. Scattering parameters are retrieved during the simulation process. The constructed design offers absorbance above 90% within a 37.89% relative bandwidth and 99.99% absorption over a vast portion of the investigated frequency range. An equivalent circuit model is presented to endorse the validity of the proposed structure. The calculated result strongly agrees with the simulated result. Symmetrical construction of the proposed unit cell reports an angular insensitivity up to a 35° oblique incidence. Post-processed simulation data confirm that the design is polarization-insensitive.

## 1. Introduction

Since the realization of metamaterials, a category of artificially-engineered material designed to exhibit unnatural electromagnetic (EM) phenomena, it has been subjected to rigorous research. Metamaterials (MMs) possess unnatural properties, such as negative permittivity (ε) and permeability (µ), an inverse Doppler effect, and so on. Negative values of ε and µ permit the energy and phase velocity of a wave to propagate in an inverse direction in the medium, thus resulting in a negative reflective index and forming left-handed material [1]. Leveraging the features of MMs, it has been investigated for different frequency ranges, i.e., GHz, THz and optical an frequency regime for most advance applications, such as sensing [2,3,4,5,6,7], satellite communication [8,9], invisibility cloaking [10,11], super lensing [12,13] and microwave-imaging [14]. Landy et al. [15] first exploited the unique characteristics of MMs, and introduced the first metamaterial perfect absorber (MMPA). MMPAs quickly allured researchers’ interest, due to the fact that they can be engineered to absorb a wide range of EM waves, and can be utilized for solar harvesting. Though the solar spectrum of the THz regime is the least-understood portion of the whole EM spectrum [16,17], research on MMPAs includes several studies on solar harvesting in the visible, infrared and ultraviolet regime [18,19,20,21].

Several polarization-insensitive MMAs have been investigated at different ranges of the THz spectrum [22,23,24,25]. Dhilon and Mittal [26] presented a dually-stacked, gold-stripped resonator that was fabricated on polyamide substrate, which yielded a result of 98% absorption and a fractional bandwidth of 12.8% for a narrow frequency range of 0.591–0.672 THz. Their illustrated data show that MMPA was insensitive to transverse electric (TE) and transverse magnetic (TM) polarization for the incident angle up to 75 degrees while maintaining absorption above 80%. A similar attempt was demonstrated by Dincer et al. [23] that showed narrowband absorptions of 99.66% at 268.82 THz in infrared, and 99.9% at 542.97 THz in visible regimes.

Many researchers have investigated multi-band MMPAs in order to achieve a greater amount of resonant absorption [19,20]. Rufangura and Sabah [18] theoretically designed a dual-band absorber with gold metallic stripes separated from the ground by a dielectric spacer in the visible frequency range. They obtained a peak absorption of 99.9% at 543 THz and 663 THz for a narrow bandwidth. Liu et al. [27] proposed an ultra-wideband absorber for solar application, but it was extremely sensitive to incident angles. Lee et al. [28] fabricated a flexible multi-band THz MMPA with gold resonator assembled on a Pyrex glass substrate, which yielded two absorption peaks at 0.98 THz and 1.55 THz, with respective absorptivities of 93% and 74%. Tao et al. [29] experimentally characterized a dual-band metamaterial absorber; their simulated design yielded absorption peaks of 85% at 1.4 THz and 94% at 3.0 THz, although the experimental result of absorption was 70% at 1.3 THz. In both of the above cases, the absorber’s bandwidths are low.

However, broadband absorbers are required for many applications, especially for solar harvesting, in terms of efficiency and economy. Several methods may be adopted to extend the bandwidth of absorbers: multiple cells of different structures can be combined into a large cell [30,31,32] to enhance its absorption range, or multiple cells can be stacked into one [33,34,35]. Li et al. [36] designed a hybrid structure of a broad-band absorber that provides relative bandwidth up to 82.9%, while Liu et al. [37] presented a tunable broadband absorber at an infrared frequency range. But these methods are constrained by fabrication processes, and dimensional limits, particularly at the THz regime, where ensuring high precision may lead to higher fabrication costs.

To overcome the limitations of lower bandwidth and lower absorption, this article proposes an ultra-wideband MA (metamaterial absorber), operating at the infrared region for solar energy harvesting. In addition, the study investigates the sensitivity of the unit cell to TE and TM polarization.

## 2. Design and Simulation Setup

**Figure 1:**
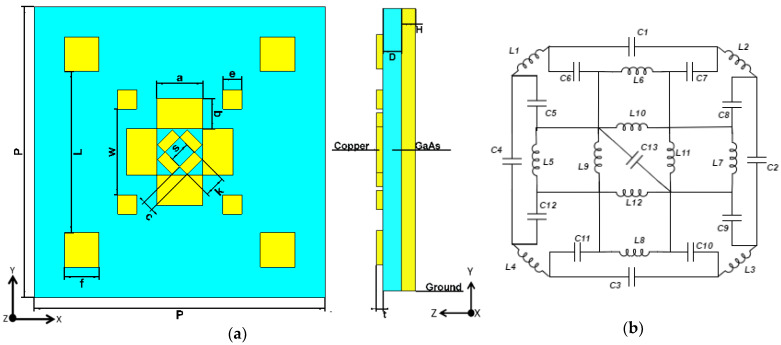
Geometry of the proposed unit cell: (**a**) top view and side view, (**b**) equivalent circuit of the proposed structure.

The absorption in metamaterial is defined as $A\left(\omega \right)=1-{\left|S_{11}\right|}^{2}-{\left|S_{21}\right|}^{2}$, where S_11_ and S_21_ are the scattering parameters (S-parameters) for reflection and transmission, respectively. The proposed MA unit cell has three layers, in which a dielectric substrate is sandwiched between two copper layers, as shown in Figure 1a. The metallic copper slabs are patched over the 66.55 nm thick dielectric GaAs substrate. A continuous metallic plate extrudes the bottom layer of the substrate. The thickness of the continuous plate, H, is maintained at a level higher than the skin depth (δ) of the material, which is defined as $\delta =\sqrt{2\rho /2\pi f\mu_{R}\mu_{0}}$, in order to ensure zero transmissions. Here, ρ indicates the conductivity of copper, µ_R_ indicates the permeability of copper and µ_0_ expresses the permeability in free space. Hence, the formula for absorption can be condensed into Equation (1).

(1) $$A\left(\omega \right)=1-{\left|S_{11}\right|}^{2}$$

An equivalent circuit model is presented to validate the design and to approximate the operating regime of the proposed absorber, as presented in Figure 1b. The equivalent inductance and capacitance can be estimated using Equations (2) and (3), leveraging techniques from [38,39,40].

(2) $$L\approx \mu_{o}D\left(\frac{\left(w+2e\right)}{2b}+\frac{\left(2a+c\right)-s}{{\left(w+2e\right)}^{2}+k^{2}}\right)$$

(3) $$C\approx \epsilon_{o}\left(\frac{\left(w+2e\right)P^{3}}{10\pi b^{2}}\right)\;\ln\frac{a}{f}$$

where, $\mu_{o}=4\pi \times 10^{-7}\;H/m$ and $\epsilon_{o}=8.854\times 10^{-12}F/m$. Equations (2) and (3) yield $1.2424\times 10^{-7}\;H$ inductance and $2.0429\times 10^{-24}\;F$ capacitance. Consequently, a resonant frequency of f=1/(2π√LC)= 315.91 THz is obtained, which is very close to the simulated result presented in the later section.

As a complex design will pose difficulties in fabrication, we have created a minimalistic design that offers horizontal and rotational symmetry.

The proposed design consists of 23.75 nm thick square- and rectangular-shaped copper patches, as Figure 1a illustrates. Copper is widely used, owing to its excellent electromagnetic properties, while costing little. As for the substrate, the loss tangent and electric permittivity of the GaAs substrate are 0.006 and ε = 12.94, respectively, which maintains the thermal conductivity of 401 Wk^−1^m^−1^. The rest of the cellular geometric parameters of the proposed absorber are mentioned in Table 1. The simulation is performed on a finite integration technique (FIT)-based ‘CST Microwave Studio’ simulation environment.

This study chose a perfect electric boundary condition (PEC) along the X plane, a perfect magnetic condition (PMC) along the Y plane, and Open (add space) boundaries along the Z plane. The wave vector k is perpendicular to the specimen, while the electric and magnetic fields are at a parallel orientation. The frequency domain solver is used during the simulation. 

Although the solar spectrum has an outstretched range of frequencies, this study focuses upon the infrared region (220–360 THz) of the solar spectrum, which covers more than 50% of the solar spectrum.

## 3. Results and Analysis

The S-parameters are retrieved in a Touchstone (SnP) format from the simulation environment, and are plotted and analyzed in MATLAB.

### 3.1. Absorption Characteristics

The absorption curve can be obtained from Equation (1) However, cross-polarization reflectivity is taken into account during the absorptivity calculation, to ensure that the unit cell is not acting as a polarization converter. The reflection coefficient contains both co-polar and cross-polar components for both TE- and TM-polarized incident waves, as expressed in Equations (4) and (5), respectively.

(4) $${\left|S_{11}\left(\omega \right)\right|}^{2}={\left|S_{TE,TE}\left(\omega \right)\right|}^{2}+{\left|S_{TE,TM}\left(\omega \right)\right|}^{2}$$

(5) $${\left|S_{11}\left(\omega \right)\right|}^{2}={\left|S_{TM,TM}\left(\omega \right)\right|}^{2}+{\left|S_{TE,TM}\left(\omega \right)\right|}^{2}$$

Here, ${\left|S_{TE,TE}\left(\omega \right)\right|}^{2}$ and ${\left|S_{TM,TM}\left(\omega \right)\right|}^{2}$ are the co-polarization reflectivity, and ${\left|S_{TE,TM}\left(\omega \right)\right|}^{2}$ is the cross-polarization reflectivity for TE- and TM-polarized incident wave. 

**Figure 2:**
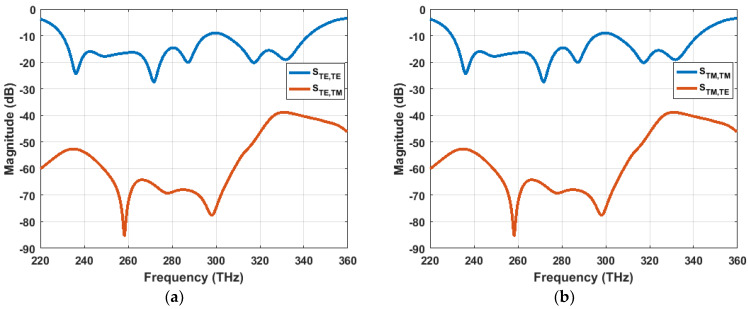
Co-polarization and cross-polarization components of the reflection coefficient (S_11_) for (**a**) transverse electric (TE)-polarized incident wave and (**b**) transverse magnetic (TM)-polarized incident wave.

As Figure 2 describes, the cross-polarization reflectivity is insignificant (almost zero in linear magnitude scale) relative to the co-polarization reflectivity, which ensure that the unit cell is not altering the incident wave’s polarization throughout the investigated frequency range.

Moreover, to analyze the absorption characteristics of the proposed MA, we have used the relative absorption bandwidth (RAB), defined as

(6) $$B_{r}=\frac{2(f_{u}-f_{l})}{\left(f_{u}+f_{l}\right)}$$

**Figure 3:**
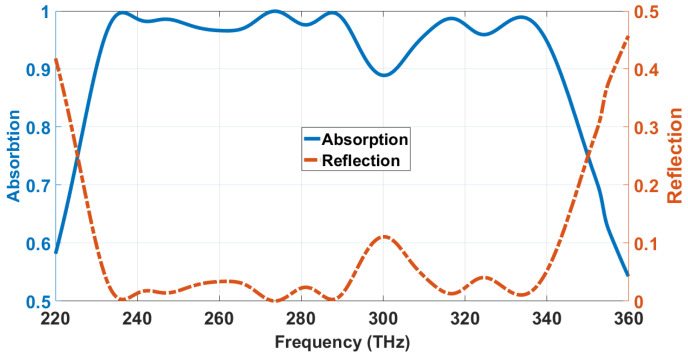
Absorption and reflection spectra of the proposed structure.

Here, *f_u_* and *f*_l_ are the upper and lower limits of the frequency range, within which the absorption is above 90%. From Equation (6), the proposed design offers absorption over 90%, maintaining a 25.602% relative bandwidth in the frequency range of 229.9–297.4 THz. It offers a 12.289% relative bandwidth, within 303.2–342.9 THz, as Figure 3 portrays. 

Therefore, the proposed MA offers total relative bandwidth, BRAB = 37.89%, with above 90% absorption, characterizing it as an ultra-wide band (UWB) absorber.

### 3.2. Polarization Independence and Angular Stabiltiy

**Figure 4:**
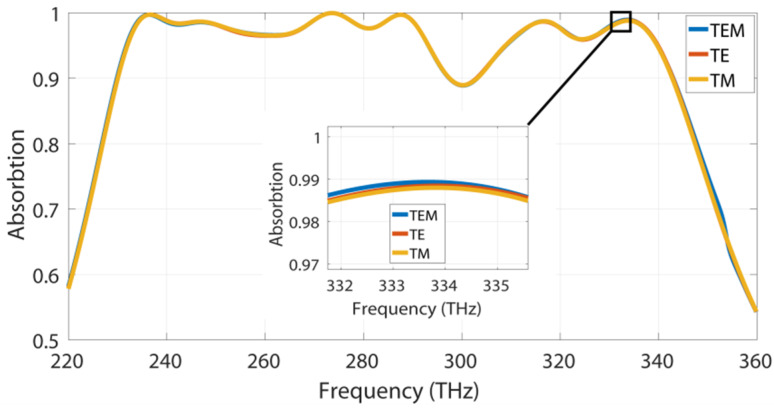
Absorption characteristics under the transverse electromagnetic mode (TEM)-, TE- and TM-polarized wave.

It is highly desirable for an ultra-wideband absorber to be polarization-independent and insensitive to any incident angle for many solar harvesting applications. We have investigated the proposed structure under the TE-, TM- and transverse electromagnetic mode (TEM)-polarized wave. The unit cell yields identical responses, regardless of the polarization mode, due to the horizontal and rotational symmetry of the proposed MA structure, as depicted in Figure 4.

**Figure 5:**
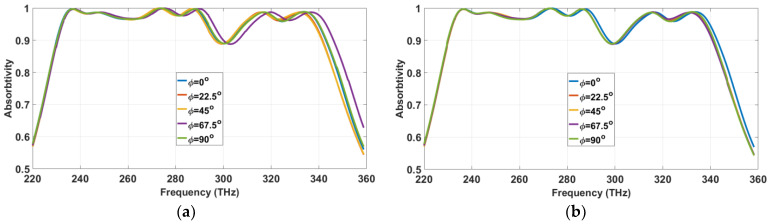
Polarization angle sensitivity at (**a**) the TE mode and (**b**) TM mode.

Varying the polarization angle (φ) from 0° to 90°, with a step size of 22.5°, is deployed to investigate polarization angle sensitivity. The unit cell shows excellent polarization angle insensitivity for the aforementioned polarized waves, as Figure 5 illustrates.

**Figure 6:**
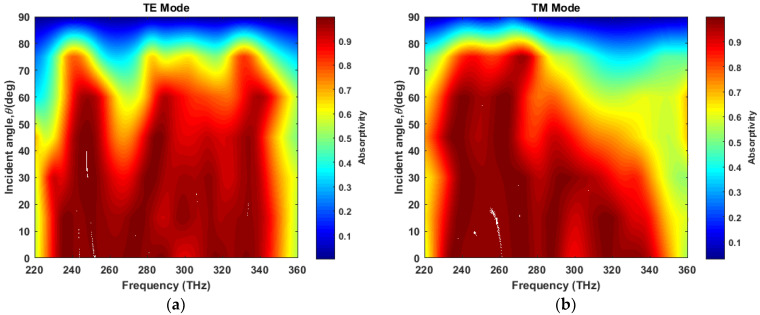
Angular sensitivity to varying incident angle, θ at (**a**) TE mode and (**b**) TM mode.

Furthermore, angular stability is investigated for the varying incident angle θ at φ = 0 degrees. At the TE mode, absorptivity degrades as the incident angle approaches 90°. Thi occurs because the decrement of the horizontal component of the E-field with the increment of the incident angle effectively reduces electrical resonance, which leads to a gradual degradation in absorptivity. However, the MA maintains high absorptivity up to 50° for a wide portion of the frequency range, and it shows excellent angular insensitivity up to 35°, as Figure 6a demonstrates. 

A similar degrading phenomenon is observed with the increment of incident angle and frequency at the TM mode (Figure 6b). However, at lower frequencies, the structure shows insensitivity for incident angles as large as 75°. Overall, the MA exhibits insensitivity to an incident angle up to 35° for the TE and TM mode.

### 3.3. Dependence on Geometric Parmeters

**Figure 7:**
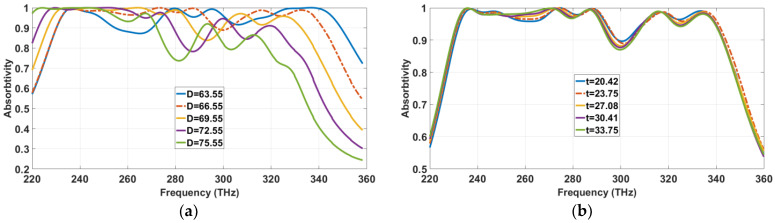
Absorption characteristics of proposed MA for different thicknesses of (**a**) dielectric substrate (D) and (**b**) metallic copper resonator (t).

This section investigates the effect of cellular parameters on the absorption characteristics of the proposed absorber. First, the thickness of GaAs substrate (D) was examined. As seen in Figure 7a, the absorption curve introduces a left shift toward lower-frequency spectra, as the thickness of the substrate increases from 63.555 nm to 75.55 nm with a step size of 3. This phenomenon occurs due to the change in the equivalent capacitance between the metallic slabs and the ground plane. The resonator layer of the proposed structure induces a series capacitance between copper patches.

As thickness D increases, the capacitance decreases for each copper slab. Consequently, the equivalent series capacitance increases for the whole metallic layer, resulting in a lower resonant frequency, which explains the left shift of absorptivity toward lower-frequency spectra.

Meanwhile, the distance between the two metallic layers also increases, which effectively reduces magnetic resonance [41], hence induces a steep fall in absorptivity.

Consequently, the thickness of the copper films (t) is altered from 22.420 nm to 33.750 nm with a step size of 3.3 nm, as Figure 7b shows. Similar to the thickness response of dielectric, the absorptivity tends to shift left (toward lower-frequencies) as the thickness (t) increases. Since the equivalent capacitance increases with thickness, its absorptivity therefore moves toward lower-frequency spectra. The MA yields an optimized absorption for t = 23.75 nm, as the dotted line indicates.

Subsequently, a parameter sweep was conducted on the lateral distances between the copper patches (denoted by parameters W and L in Figure 1a), in order to investigate the effect of gap capacitance between the metallic copper plates.

**Figure 8:**
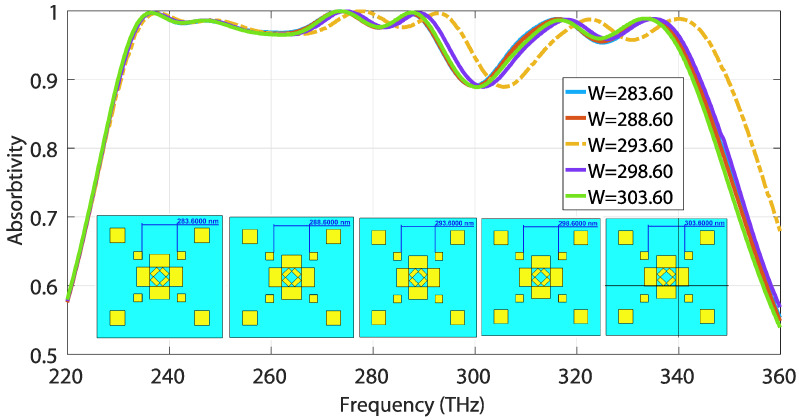
Absorption sensitivity to the lateral distance denoted by W.

The absorption characteristics remain almost identical over the whole sweep, from W = 280 to W = 303 nm with a step size of 5 nm, as depicted in Figure 8. However, a trend of decreasing bandwidth is observed as the distance W increases. The yellow-dotted absorption curve for W = 293.60 shows an exceptionally higher relative absorption bandwidth than the others, while maintaining a decent absorption rate over the frequency spectrum. 

**Figure 9:**
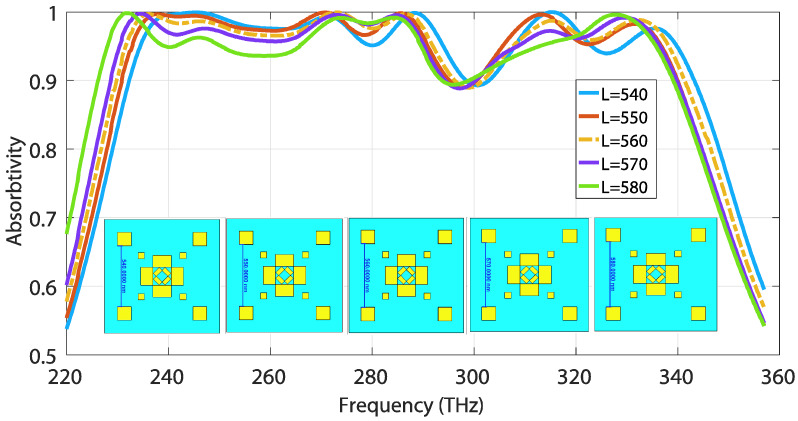
Absorption sensitivity to the lateral distance denoted by L.

As for lateral distance L, the absorption rate noticeably declines with the increment of L with a step size of 10 nm, as depicted in Figure 9. The absorption curve tends to shift left (toward lower-frequency regions) as the capacitance between the gap drops with distance.

The relative absorption bandwidth at over 90% absorption remains almost identical throughout the sweep, yet absorption above 95% dramatically drops with the escalation of distance. The optimized absorption curve is indicated by the dotted line for L = 560 nm. The structure notably exhibits a similar frequency shifting trend for both varying lateral distances, W and L. The reason behind this phenomenon is that, as the distance increases, the inter-metallic gap capacitance of each slab decreases; consequently, the equivalent series capacitance of the whole metallic layer intensifies, thereby introducing a shift toward lower resonant frequency spectra, and vice-versa.

### 3.4. Mechanism of Absorption

To realize the nature of the absorption behavior, the surface current and the electric field (E-field) are analyzed at two resonant frequencies 273.76 THz and 333.72 THz, with peak absorption. Perfect adsorption was achieved at resonance frequencies due to the simultaneous occurrence of electric resonance and magnetic resonance—i.e., when the electric and magnetic responses of the absorber match with that of the incident wave.

**Figure 10:**
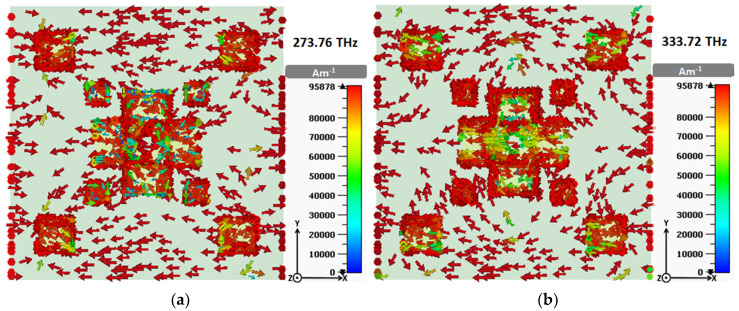
Surface current distribution of the metallic layer at (**a**) 273.76 THz and (**b**) 333.72 THz.

It can be observed from Figure 10 that the MA induces a high concentration of circulating current at the edge of the copper resonators. A closer examination reveals the existence of parallel and anti-parallel surface currents moving around the copper slabs, which introduces electric resonance to the absorber. Meanwhile, the circulating current produces magnetic resonance [18].

**Figure 11:**
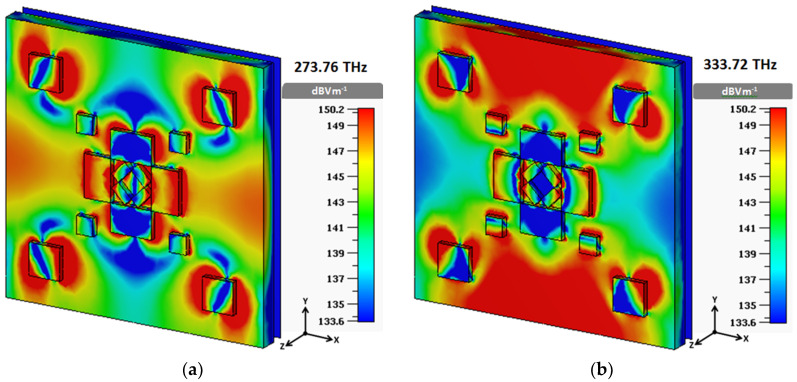
E-field distribution at (**a**) 273.76 THz and (**b**) 333.72 THz.

Another approach to realizing the absorption process is to analyze the E-field distribution at resonant frequencies. In Figure 11, it seems that the E-field is denser at the edges of copper slabs, such that it acts like electric dipoles, which are accountable for electrical resonance. Additionally, the strong dipole-like E-field creates surface charges that oscillate with the electric field, thus resulting in magnetic resonance [18]. 

**Figure 12:**
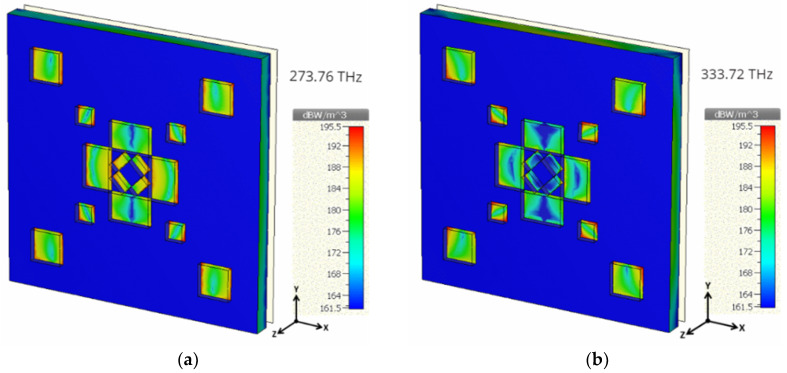
Power loss distribution at (**a**) 273.76 THz and (**b**) 333.72 THz.

Furthermore, power loss distributions at similar resonant frequencies were investigated in order to understand the trend of absorption in dielectric and the metallic layer. It can be concluded from Figure 12 that the copper resonators are mostly accountable for the wideband absorption, even though GaAs has a low loss tangent (0.0006). The reason behind this is, the metallic layer tends to be lossier than the dielectric at the infrared frequency regime [41], which holds the metallic layer more responsible for wideband absorption. Synthesis of the proposed structure can be performed through an extensively used photolithography technique. However, photolithography requires a complicated procedure, including photo-masking of the samples. Murata et al. [42] introduced a super-fine ink-jet (SIJ) printer, which is capable of fabricating three-dimensional nano-structures with an accuracy analogous to photolithography. This method may be adopted to avoid copper-oxide formation during the synthesis of the structure.

The proposed absorber is compared to absorbers from several recent studies comprising similar features; i.e., ultra-wideband absorption and operating at infrared frequency spectra. The comparison data in Table 2 show the proposed absorber has a significantly larger fractional and absolute bandwidth for long frequency range. The proposed absorber is constructed with comparatively low-cost materials, and offers polarization insensitivity while maintaining angular stability up to 35°. With all these characteristics, the proposed absorber is a considerable candidate for solar harvesting applications.

## 4. Conclusions

Metamaterial has been utilized to enhance the efficiency of solar energy harvesting. This study designed an ultra-broadband absorber in the infrared regime. The three-layered MA is designed with a copper resonator, where a GaAs substrate is sandwiched between the resonator and the ground layer. An equivalent circuit model is presented in order to validate the design and to understand resonant behavior. Although the solar spectrum is outstretched for a wide range of frequencies, the proposed absorber operates at the infrared regime, which covers more than 50% of the solar spectrum. By the proper tuning of geometric parameters, we obtained an optimized structure that provides near-unity absorption over an ultra-range of infrared frequency regime. The trends of absorption quality are also observed with the change in parameters. The proposed design offers absorption above 90% within a relative bandwidth of 37.89%, which characterizes the design as an ultra-wideband absorber. The proposed unit cell also offers near-perfect absorption: 99.99% absorption for a vast portion of the frequency range. In addition, the absorber also offers angular stability for incident angles up to 35°, as well as exhibiting excellent insensitivity to polarization, due to the horizontal and rotational symmetry of the proposed absorber for TE-, TM- and TEM-polarized waves. All these features make the design a potential candidate for solar harvesting applications.

## Figures and Tables

**Table 1:** Structural parameters for proposed unit cell construction.

Parameter	Value (nm)
A	160
B	105.6
C	36.4
K	72.8
E	66.4
F	120
T	23.7
P	1010
S	72
H	50
D	66.5
L	560
W	298.6

**Table 2:** Comparison with recent studies of ultra-wideband absorbers.

Ref.	Frequency Range THz	Fractional Bandwidth A(ω) > 90%	Absolute Bandwidth THz	Material Used	Dimensionsz λ (μm)	Polarization insensitive	Angular Stability	Validation
[43]	136–374	65.8%	168	Au, Ti, SiO_2_	0.27λ × 0.27λ × 0.13λ	Yes	θ < 40°	Simulated
[44]	293–750	72.2% (A(ω) < 90%)	331.42 (A(ω) < 90%)	Al_2_O_3_, Ge_2_Sb_2_Te, Al	0.4λ × 0.4λ × 0.21λ	Yes	θ = 0°	Simulated
[45]	330–750	56%	291.46	Mn, Al_2_O_3_	0.6λ × 0.6λ × 0.3λ	No	θ ≤ 20°	Measured
[46]	0.1–3.0	187.09%	2.9	Cu, Si	5λ × 5λ × 0.15λ	Yes	θ = 0°	Measured
[47]	21.43–37.47	47.61%	14.41	Ti, Si	0.27λ × 0.27λ × 0.05λ	No	θ = 0°	Simulated
[48]	24.98–28.82	3.7%	1.07	In_2_SnO_5_, ZnS	0.86λ × 0.86λ × 0.055λ	Yes	θ ≤ 50°	Measured
This work	220–360	37.89%	108	Cu, GaAs	0.77λ × 0.77λ × 0.11λ	Yes	θ ≤ 35°	Simulated
